# Direct Effects of Capsaicin on Voltage-Dependent Calcium Channels of Mammalian Skeletal Muscle

**DOI:** 10.3390/biom16010135

**Published:** 2026-01-13

**Authors:** Dmytro Isaev, Tatiana Prytkova, Badarunnisa Mohamed, Mohamed Omar Mahgoub, Keun-Hang Susan Yang, Murat Oz

**Affiliations:** 1 Department of Cellular Membranology, Bogomoletz Institute of Physiology, 01024 Kiev, Ukraine; 2 Department of Biological Sciences, Schmid College of Science and Technology, Chapman University, One University Drive, Orange, CA 92866, USA; 3 Research Core Facility, Faculty of Medicine, Kuwait University, Safat 13110, Kuwait; 4 Department of Health Sciences, College of Natural and Health Sciences, Zayed University, Abu Dhabi 144534, United Arab Emirates; 5 Department of Pharmacology and Therapeutics, College of Pharmacy, Kuwait University, Safat 13110, Kuwait

**Keywords:** calcium channels, capsaicin, T-tubule, skeletal muscle

## Abstract

Capsaicin, a naturally occurring polyphenol, is known to affect energy expenditure and muscle fatigue and modulate contractions in skeletal muscle. The L-type Ca^2+^ channels are known to be an important ion channel involved in the various muscle functions and the effect of capsaicin on the skeletal L-type Ca^2+^ channels is currently unknown. In this study, the effects of capsaicin and capsaicin analogs on depolarization-induced Ca^2+^ effluxes through L-type Ca^2+^ channels in transverse tubule membranes from rabbit skeletal muscle and L-type Ca^2+^ currents recorded using the whole-cell patch clamp technique in rat myotubes were examined. Capsaicin, in the concentration range of 3–100 µM, inhibited depolarization-induced Ca^2+^ effluxes. The effect of capsaicin was not reversed by TRPV1 antagonist SB-366791 (10 µM). While vanilloids (30 µM) including vanillin, vanillyl alcohol, and vanillylamine were ineffective, other capsaicinoids (30 µM) including dihydrocapsaicin, nonivamide, and nordihydrocapsaicin significantly inhibited Ca^2+^ effluxes, suggesting that hydrocarbon chains are required for inhibition. In rat myotubes, capsaicin inhibited L-type Ca^2+^ currents with an IC_50_ value of 27.2 μM in the presence of SB-366791. Furthermore, in docking studies and molecular dynamic simulations, capsaicinoids with an aliphatic tail showed stronger binding and stable bent conformations in CaV1.1, forming hydrogen bonds with Ser1011 and Thr935 and hydrophobic/π–alkyl contacts with Phe1008, Ile1052, Met1366, and Ala1369, resembling the binding mode of amlodipine. In conclusion, the results indicate that the function of L-type Ca^2+^ channels in mammalian skeletal muscle was inhibited by capsaicin and capsaicin analogs in a TRPV1-independent manner.

## 1. Introduction

In skeletal muscle, L-type voltage-dependent calcium channels (VDCCs, CaV1.1) are mainly located in the transverse (T)-tubule system and have dual functions in muscle contractions as voltage sensors and performing channel functions [1,2]. CaV1.1 serves primarily as a voltage sensor and translates the excitation to contraction process [1]. Excitation–contraction coupling in skeletal muscle does not require the channel function of VDCC [3,4]. However, L-type VDCCs have been suggested to play functional roles in excitation-coupled calcium entry, defined as the entry of extracellular Ca^2+^ into skeletal muscle during sustained depolarizations or long-lasting tetanic stimulations [5]. In previous studies, it has been proposed that the entrance of Ca^2+^ through L-type Ca^2+^ channels occurs during tetanic stimulations [6,7,8,9] and contributes to the replenishment of the sarcoplasmic reticulum Ca^2+^ stores, and provides support to maintain muscle performance during prolonged contractions [10]. In addition, using non-conducting CaV1.1 mutants [8] and CaV1.1 splice variant models [11], it has been shown that reducing Ca^2+^ influx through L-type VDCCs significantly affected fiber-type specification [12] and Ca^2+^-dependent fatty acid metabolism [13] as well as neuromuscular junction formation and clustering patterns of nicotinic acetylcholine receptors during embryonic development [14,15].

The naturally occurring alkaloid capsaicin, which is found in chili pepper, is known to be responsible for the hot and strong flavor of this plant. In vitro and in vivo studies have demonstrated that capsaicin exerts several pharmacological effects, including analgesia, anti-inflammation, anti-obesity, anti-cancer, and antioxidant actions [16,17]. In skeletal muscle, capsaicin has been shown to modulate energy expenditure, muscle fatigue, contractions, and intracellular Ca^2+^ homeostasis [18,19]. The transient receptor potential vanilloid subfamily member 1 (TRPV1) is the major receptor mediating most of the pharmacological actions of capsaicin. However, several earlier studies report that capsaicin, in addition to the TRPV1 receptor, modulates the functions of neurotransmitter receptors and ion channels [20]. At low to mid µM ranges, capsaicin has been reported to indirectly affect functions of various ion channels through TRPV1-mediated Ca^2+^ increases. In addition, at these concentration ranges, capsaicin also directly affects the functions of ligand-gated ion channels [21,22], as well as voltage-gated Ca^2+^, K^+^, and Na^+^ channels [23,24] and transporters involved in cellular excitability and muscle contractions [20].

The Ca^2+^ flux measurements through skeletal muscle T-tubule membranes have been used in previous studies to examine the functional characteristics of L-type VDCCs [25,26,27]. As T-tubule membranes are arranged as sealed, inside-out vesicles, which do not include intracellular organelles [26,27], L-type VDCCs in this preparation can be studied in their native membranes without interference by intracellular events.

**Figure 1:**
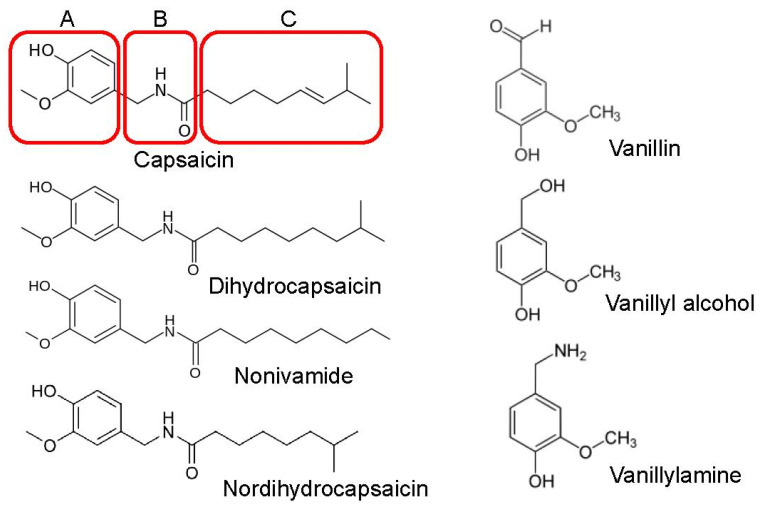
The chemical structure of capsaicin, the main component of chili pepper, and its three major regions, namely A (aromatic head), B (amide linkage), and C (hydrophobic tail), are shown in various boxes. Chemical structures of capsaicin, nonivamide, dihydrocapsaicin, nordihydrocapsaicin, vanillin, vanillyl alcohol, and vanillylamine are presented in the figure.

Structurally, capsaicinoids, including those used in this study, belong to a class of compounds known as vanilloids. As shown in Figure 1, a vanillyl (methylcatechol) head group (A-region) and an aliphatic tail (hydrophobic-C-region) linked by a central amide bond (B-region) primarily constitute the chemical structure of capsaicinoids. In this study we compared the effects of capsaicinoids including dihydrocapsaicin, nonivamide, and nordihydrocapsaicin, as well as vanillin derivatives such as vanillin, vanillyl alcohol, and vanillylamine, on Ca^2+^ effluxes through L-type VDCCs in T-tubule membranes, and L-type Ca^2+^ currents in rat skeletal muscle myotubes.

## 2. Materials and Methods

### 2.1. Preparation of Transverse Tubule Membranes

Four-to-six-week-old New Zealand white rabbits (1–1.5 kg) were decapitated, and microsomal membranes were prepared from the hind and back muscles. T-tubules were isolated using sucrose gradient centrifugation following previously described protocols [25,26,28]. T-tubule membranes were resuspended and equilibrated in low potassium buffer (10 mM HEPES-Tris, pH: 7.4, 145 mM choline chloride, 5 mM potassium gluconate, 0.02% NaN_3_), and kept at −86 °C. Prior to experiments, the vesicles underwent several freeze–thaw cycles to equilibrate intracellular and extracellular ion concentrations [26].

### 2.2. ^45^Ca^2+^ Efflux Assay

T-tubule membranes (approximately 0.4 mg/mL) were loaded with ^45^Ca^2+^ by adding an equal half volume of isotopically diluted ^45^CaCl_2_ solution to the same buffer, yielding a final concentration of 5 mM total Ca^2+^ containing 50 μCi/mL ^45^Ca^2+^ (PerkinElmer, Springfield, IL, USA). Calcium loading was achieved through two freeze–thaw cycles, after which suspensions were kept on ice and used within 1–2 h. Voltage-dependent ^45^Ca^2+^ efflux was assessed using a two-step filtration assay [25,26]. For efflux measurements, 25 μL of loaded membranes were diluted in 975 μL of high-potassium buffer (10 mM HEPES-Tris, pH 7.4, 120 mM potassium gluconate, 30 mM choline chloride, 0.133 mM EGTA) containing 0.1 μM valinomycin and the appropriate drug where indicated. The initial dilution mimics a cell’s resting state by creating an external (quasi-intracellular) negative membrane potential at −80 mV and lowering free Ca^2+^ outside the vesicle (equivalent to intracellular Ca^2+^ in an inside-out vesicle) to <100 nM. Following a 10 min incubation at room temperature (18–21 °C), 0.9 mL of the suspension was transferred onto a GF/C filter (pre-equilibrated in the same buffer and dried under a vacuum). Excess buffer was removed under vacuum, after which 1 mL of depolarizing buffer (10 mM HEPES-Tris pH 7.4, 5 mM potassium gluconate, 145 mM choline chloride, 0.133 mM EGTA, 0.1 mM valinomycin) was applied. This two-step protocol (F) is referred to as 5-120-5 mM K^+^ in the text. Control experiments (C) used dilution buffers at a constant K+ concentration (5-5-5 mM K^+^). Efflux on the filter proceeded for 15 s, after which the extravesicular solution was rapidly removed by two successive washes of 5 mL of a “stop” solution (10 mM HEPES-Tris pH 7.4, 145 mM choline chloride, 5 mM potassium gluconate, 0.5 mM LaCl_3_, 30 mM sucrose). Filters containing membrane vesicles were then dried, extracted with 5 mL of HydrofluorTM scintillation fluid (National Diagnostics, Fort Lauderdale, FL, USA), and counted for ^45^Ca^2+^.

Capsaicin, other capsaicin analogs, vanillyl and its derivatives, SB-366791, and all other reagents were obtained from Sigma (St. Louis, MO, USA), while capsazepine was purchased from Tocris Cookson (Bristol, UK). Stock solutions of capsaicin and its analogs were dissolved in dimethylsulfoxide (DMSO), with all samples, including controls, containing a final DMSO concentration of less than 0.2%. Drugs and other agents were applied in ice-cold buffer solution and incubated with ^45^Ca^2+^-loaded vesicles prior to experiments.

### 2.3. Data Analysis

Results of efflux experiments are presented as the arithmetic means ± standard errors of the means (S.E.M.), with the number of determinations (*n*) shown above each bar. For each experiment, the quantity of ^45^Ca^2+^ retained by the vesicles under control conditions (i.e., without membrane potential alteration changes) was first measured. The mean counts per minute (cpm) from control samples was calculated and normalized to 100%. To determine the S.E.M values, cpm data from individual determinations were also normalized to the mean control value. All experimental data obtained under other conditions are presented as a percentage relative to these control values. Paired *t*-tests, Student’s *t*-tests, or analysis of variance (ANOVA), followed by post hoc Bonferroni tests were used to statistically evaluate the data. The Origin^TM^ (Microcal Software, Version 8.5, OriginLab Corporation, Northampton, MA, USA) software was used for data analysis and calculations. Logistic equation and non-linear decay or linear curve-fitting functions of the software were employed for data analysis.

### 2.4. Primary Cultures of Rat Skeletal Muscle Cells

Hind limb muscles of Sprague-Dawley rats (days 1–2) were dissected. Procedures for cell dissociation and culture were as described previously [29,30,31] with minor modifications. Briefly, muscles from one to three neonatal pups were dissected in sterile cold Hanks’ balanced salt (Ca^2+^- and Mg^2+^-free) solution (HBSS: 137 mM NaCl; 5.4 mM KCl; 0.25 mM Na_2_HPO_4_; 0.44 mM KH_2_PO_4_; 4.2 mM NaHCO_3_). After 20 min incubation at 37 °C in HBSS supplemented with 0.25% trypsin (*w*/*v*), minced tissue was triturated with Pasteur pipettes. The resulting suspension was passed through a Nylon-mesh cell strainer (Invitrogen, Carlsbad, CA, USA) to remove residual tissue fragments. The flow-through was collected and centrifuged (10 min, 0–4 °C, 1400 rpm). The cell pellet was resuspended in Dulbecco’s modified Eagle’s medium (DMEM) containing 10% fetal calf serum, 200 mM glutamine, and 12.5 units/mL penicillin-streptomycin (plating medium). Cells were plated in 35 mm plastic dishes on glass coverslips coated with 1% gelatin and incubated (in 5% CO_2_, water-saturated air) at 37 °C. After seventy-two hours, the medium was replaced with differentiation medium (DMEM plus 5% inactivated horse serum). To induce the formation of rounded myotubes (myoballs), the culture medium was supplemented with Colchicine (Sigma; 30 nM) three days after cell plating when mature myotubes began to appear. Penicillin-G (100 U/mL, Sigma) and streptomycin (50 mg/mL, Sigma) were included in all culture media. After the addition of colchicine, cells were used within two days, since the major calcium channel subtype is L-type VDCCs during this period [29,31].

### 2.5. Electrophysiological Recordings

Whole-cell patch-clamp recordings were performed using an Axopatch 200B amplifier (Axon Instruments-Molecular Devices, Sunnyvale, CA, USA) as previously described [30]. Micropipettes were fabricated from borosilicate glass capillaries and exhibited tip resistance of 3–5 MΩ. The internal (pipette) solution contained (in mM) 140 Cs-aspartate; 5 Mg-aspartate, 10 Cs_2_EGTA, and 10 N-(2-hydroxyethyl) piperazine-N′-(2-ethanesulfonic acid (HEPES); pH was adjusted to 7.4 with CsOH. The external recording solution used for Ca^2+^ current measurements consisted of (in mM) 145 TEA (tetraethylammonium hydroxide)-Br, 10 CaCl_2_, 10 HEPES, and 0.001 tetrodotoxin, adjusted to pH 7.4 using CsOH. Whole-cell currents were recorded and low-pass filtered at 5 kHz using pClamp 6.04 software (Axon Instruments). Analog-to-digital conversion was achieved via Digidata 1200 (Axon Instruments). Data analysis, integration, and kinetic analysis of current traces were performed with Origin^TM^ version 8.5 (OriginLab Corporation, Northampton, MA, USA).

### 2.6. Molecular Docking and System Preparation

The structure of the rabbit voltage-gated calcium channel CaV1.1 (PDB ID: JPX.pdb) [32] was retrieved from the Protein Data Bank. Crystallographic water molecules, ions, and non-standard residues were removed, and missing atoms were rebuilt. Disulfide bonds were verified, and protonation states of titratable residues were assigned to correspond to physiological pH 7.4.

The three-dimensional structures of seven compounds (capsaicin, dihydrocapsaicin, nonivamide, nordihydrocapsaicin, vanillin, vanillyl alcohol, and vanillylamine) were retrieved from the PubChem database [33], energy-minimized, and arranged for docking using AutoDock Tools (version 1.5.7) [34]. Molecular docking calculations were carried out using AutoDock Vina (version 2.0) [35], with the grid box centered on the CaV1.1 binding site occupied by amlodipine in PDB entry 7JPX. For each ligand, the conformation with the least predicted binding free energy and the most favorable orientation within the pocket was selected for subsequent molecular dynamics (MD) simulations.

### 2.7. Membrane System Construction

The receptor–ligand complexes were embedded into a POPC/cholesterol (9:1) lipid bilayer using PACKMOL-Memgen as described by [36]. The membrane composition and thickness were chosen to mimic a typical mammalian plasma membrane. The system was solvated using explicit molecules of TIP3P water, with a 15 Å buffer above and below the membrane, and neutralized with Na^+^ and Cl^−^ ions at a physiological concentration of 0.15 M. The assembled bilayer–protein complex was aligned along the membrane normal and subjected to visual inspection to ensure correct orientation and absence of steric clashes between lipids and the transmembrane regions.

### 2.8. Molecular Dynamics Simulations

All molecular dynamics simulations were carried out using AMBER22 [37] and AmberTools [38], with the ff19SB force field for the protein, Lipid21 for the lipids, GAFF2 for ligands, and TIP3P for water molecules. After topology generation, the system underwent a multistage equilibration protocol adapted from the AMBER membrane protein tutorial. Energy minimization was performed in two phases: an initial minimization with restraints on protein and ligand heavy atoms, followed by an unrestrained minimization to relax the entire system.

The system was gradually heated from 0 K to 303 K under the constant-volume ensemble (NVT) over 200 ps, using harmonic restraints on the protein backbone and ligand atoms. This was followed by equilibration under the constant-pressure ensemble (NPT) for 2 ns with progressively reduced restraints. A subsequent 1 ns equilibration was carried out with weak restraints on the Cα atoms of the receptor to stabilize the membrane–protein interface. Production simulations were conducted for 500 ns at 303 K and 1 atm in the NPT ensemble. The SHAKE algorithm was applied to constrain bonds involving hydrogen atoms, and hydrogen mass repartitioning allowed for a 4-fs integration timestep. Long-range electrostatics were treated with the Particle Mesh Ewald (PME) method, and a 10 Å cutoff was applied for nonbonded interactions. The Langevin thermostat was used to maintain temperature stability throughout the simulations.

### 2.9. Trajectory Analysis

Trajectory analyses were performed using CPPTRAJ [39]. All trajectories were imaged and centered to correct for periodic boundary artifacts. Structural stability was assessed through root-mean-square deviation (RMSD) and root-mean-square fluctuation (RMSF) analyses of the receptor and ligands. The number and persistence of hydrogen bonds, as well as key noncovalent interactions, were quantified to evaluate binding stability. Visualization of representative structures and receptor–ligand interactions were performed using PyMOL (version 3) and BIOVIA Discovery Studio Visualizer (2022 Client 22.1).

## 3. Results

### 3.1. Effect of Capsaicin on Depolarization-Induced Ca^2+^ Fluxes

**Figure 2:**
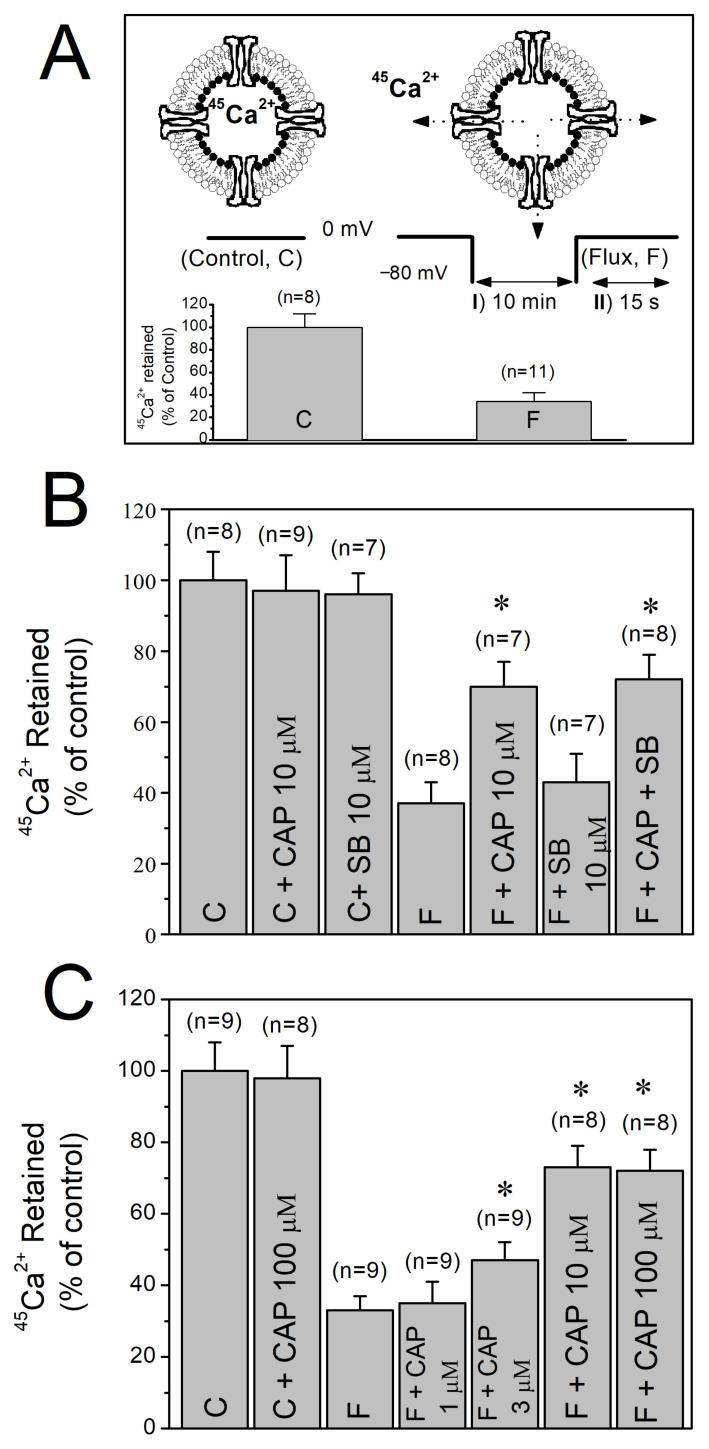
The effects of capsaicin on high K^+^-evoked ^45^Ca^2+^ efflux through T-tubule membranes. (**A**) Isolated, inside-out T-tubule membranes are shown schematically. I and II, respectively, represent the two-step protocol employed to evoke ^45^Ca^2+^ fluxes. The amounts of ^45^Ca^2+^ in vesicles measured before (control; C) and after (flux; F) depolarizations are shown in the inset as percentage bars. (**B**) Effects of capsaicin and SB-366791 on depolarization-induced ^45^Ca^2+^ effluxes. (**C**) Concentration dependent effect of capsaicin on high K^+^-induced ^45^Ca^2+^ effluxes. Number of trials (n) is shown at the top of each column. Vertical lines at the top of the columns indicate the S.E.M. * denotes statistical significance at the level of *p* < 0.05 when compared to the efflux (F) group. C, control conditions; F, efflux conditions; CAP, capsaicin; SB, SB-366791.

The outline of the two-step protocol and the orientation of the isolated T-tubule vesicles are illustrated in Figure 2A. Under control conditions where the K^+^ concentration remained constant at 5 mM throughout the flux assay and the membrane potential was not changed (5-5-5 mM K^+^), no measurable efflux of ^45^Ca^2+^ from the vesicles was observed (C in inset to Figure 2A). In contrast, repolarization induced by addition of high external K^+^ and subsequent exposure to depolarizing solution for 15 sec (5-120-5 mM K^+^, F in inset to Figure 2A) reduced the ^45^Ca^2+^ content of the vesicles to 30–35% of the control levels.

Preincubation with capsaicin (3–100 μM) for 10 min significantly inhibited the high K^+^-evoked ^45^Ca^2+^ efflux responses without altering basal ^45^Ca^2+^ content under control conditions (*p *< 0.05, ANOVA and post hoc Bonferroni test, *n* = 8–9, Figure 2B). The inhibitory action of capsaicin was not reversed by the TRPV1 antagonist SB-366791 (10 µM). Interestingly, commonly used TRPV1 antagonist, capsazepine (10 µM), alone significantly inhibited Ca^2+^ effluxes (data not shown, *p *< 0.05, ANOVA and post hoc Bonferroni test, *n* = 6–8). In the concentration range of 1–100 µM, the inhibitory effect of capsaicin was concentration-dependent (Figure 2C).

**Figure 3:**
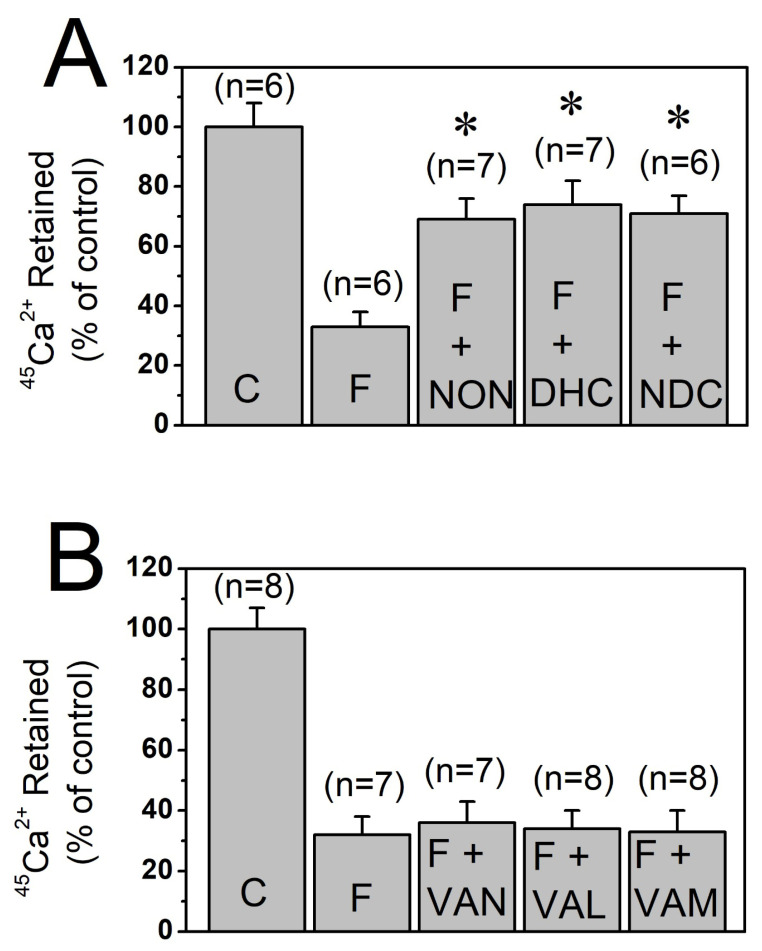
Effects of different vanilloids and capsaicin analogs on high K^+^-evoked ^45^Ca^2+^ effluxes through T-tubule membranes. (**A**) Effects of 30 µM nonivamide, dihydrocapsaicin, and nordihydrocapsaicin on high K^+^-evoked ^45^Ca^2+^ effluxes. (**B**) Effects of 30 μM vanillyl alcohol, vanillin, and vanillylamine on ^45^Ca^2+^ efflux responses. Number of trials (n) is shown at the top of each column. Vertical lines at the top of the columns indicate the S.E.M. * denotes statistical significance at the level of *p* < 0.05 when compared to the efflux (F) group. C, control conditions; F, efflux conditions; NON, nonivamide; DHC, dihydrocapsaicin; NDC, nordihydrocapsaicin; VAN, vanillin; VAL, vanillyl alcohol; and VAM, vanillylamine.

Capsaicin belongs to the vanilloid family of compounds and features a vanillyl (methylcatechol) head group (A-region) connected via a central amide bond (B-region) to a hydrophobic aliphatic tail (C-region) as illustrated in Figure 1. Incubation of T-tubule membrane preparations with 30 μM of nonivamide, dihydrocapsaicin, and nordihydrocapsaicin significantly inhibited the vesicular ^45^Ca^2+^ effluxes without altering the vesicular ^45^Ca^2+^ content under control conditions (*p* < 0.05, ANOVA with Bonferroni post hoc test, *n* = 6–7, Figure 3A). In contrast, treatment with 30 μM vanillin, vanillyl alcohol, and vanillylamine for 10 min produced no significant change in ^45^Ca^2+^ levels under either control or flux conditions (Figure 3B).

### 3.2. The Effects of Capsaicin on the L-Type Calcium Currents Recorded in Rat Skeletal Muscle Myotubes

**Figure 4:**
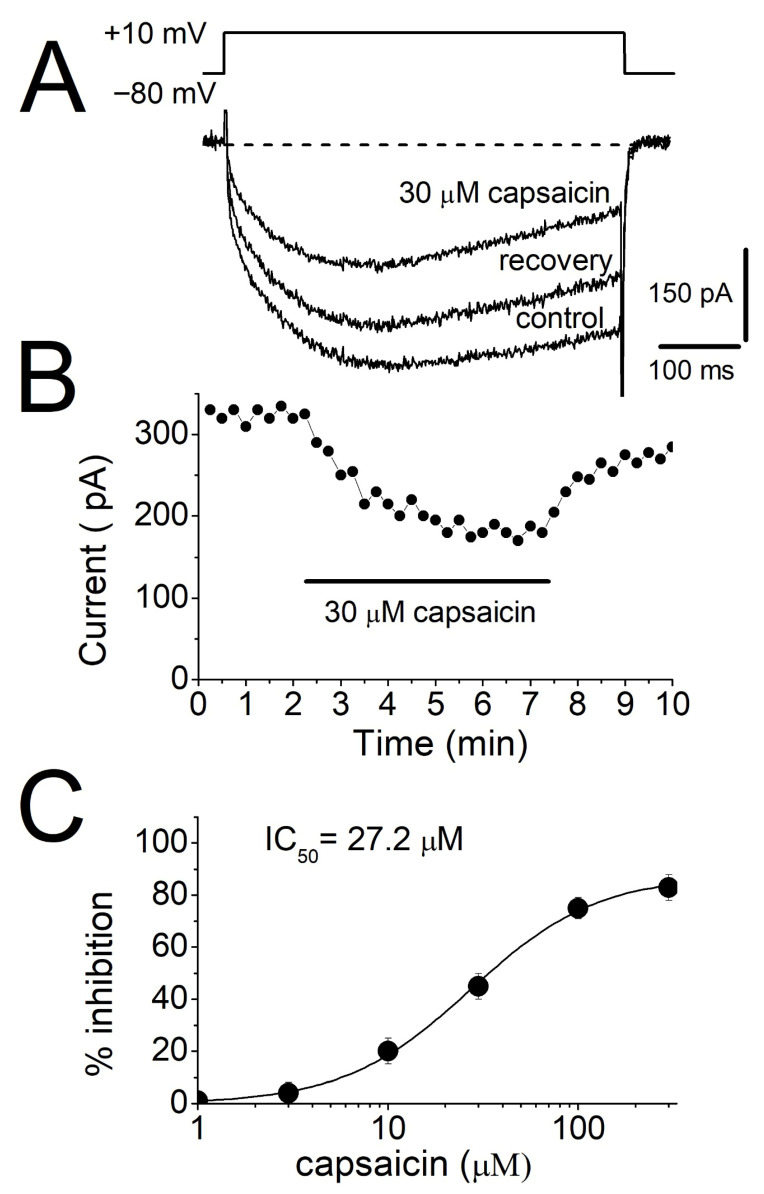
The effect of capsaicin on Ca^2+^ currents mediated by L-type voltage-dependent calcium channels in rat myotubes. (**A**) Calcium currents recorded using the voltage-clamp mode of the whole-cell patch clamp technique were inhibited by capsaicin. Current traces were shown before (control) and 5 min after 30 μM capsaicin application were presented. Every 15 s, depolarizing test pulses to +10 mV for 0.5 s were induced from a holding potential of −80 mV. (**B**) Time course of capsaicin inhibition of L-type voltage-dependent calcium channels. After two minutes of baseline recording, extracellular solution containing 30 μM capsaicin was applied for 5 min (horizontal bar). (**C**) Effects of increased capsaicin concentration on maximum amplitudes of calcium currents. The IC_50_ value was obtained from non-linear regression fits of the data points (*n* = 4–6 cells).

The effects of capsaicin on L-type voltage-dependent Ca^2+^ currents were examined in rat myotubes utilizing whole-cell configuration of patch-clamp technique. Cells were voltage-clamped at a holding potential of −80 mV, and inward Ca^2+^ currents were elicited by depolarizing test pulses to +10 mV (500 ms duration) applied at 15 s intervals. These protocols evoked slowly activating inward currents which were completely blocked by 10 μM Isradipine (Supplemental Figure S1, *n* = 4). To exclude TRPV1-mediated effects, 10 µM of selective TRPV1 receptor antagonist, SB-366791 [40], was included in perfusion solution, since TRPVI receptors are expressed in skeletal muscle fibers [41,42]. Under these conditions, extracellular application of capsaicin (30 μM) for 5 min produced a significant inhibition of L-type Ca^2+^ currents (Figure 4A). The inhibitory effect developed gradually during capsaicin exposure, reaching a steady-state reduction within approximately 5 min, and exhibited partial reversibility upon washout (Figure 4B). Concentration-response analysis revealed that capsaicin produced a dose-dependent reduction in the amplitude of peak L-type Ca^2+^ currents with an IC_50_ value of 27.2 μM (Figure 4C).

**Figure 5:**
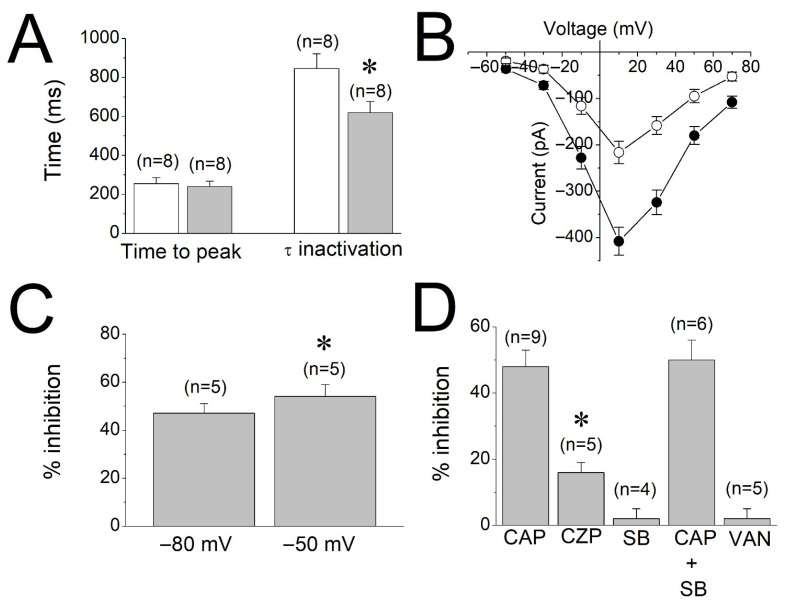
Effect of capsaicin on the kinetics and the current–voltage relationship of calcium currents, and summary of effects of test compounds on calcium currents. (**A**) Effect of capsaicin on L-type Ca^2+^ current kinetics in rat myotubes. Means and the S.E.M. of the time to reach peak amplitude and inactivation time constant (τ) are shown before (control, white bars) and five min following 30 μM capsaicin (gray bars) application (*p* < 0.05; ANOVA). (**B**) Current–voltage relationship of Ca^2+^ currents in the absence (filled circles) and presence (open circles) of 30 μM capsaicin is presented. The 500 ms depolarizing voltage steps from a holding potential of −80 mV to command potentials between −50 and +70 mV were used to evoke currents. The data points are the mean and the S.E.M. values from five cells. (**C**) The effects of 30 µM capsaicin on Ca^2+^ currents activated by depolarizations to +10 mV from holding potentials of −80 mV or −50 mV (*n* = 5 cells). (**D**) The effects of capsaicin and TRPV1 antagonists on the maximal amplitudes of L-type Ca^2+^ currents in rat myotubes. CAP: capsaicin; CPZ: capsazepine; SB: SB-366791; VAN: vanillin. * denotes statistical significance at the level of *p* < 0.05 when compared to the controls.

In the presence of capsaicin (30 µM), the time to peak of L-type Ca^2+^ currents (256 ± 32 ms for controls versus 242 ± 29 ms in capsaicin; paired *t*-test, *p* > 0.05) was not significantly changed (Figure 5A). However, the means of inactivation time constants before (control) and after capsaicin application were significantly reduced from 847 ± 76 ms for controls to 619 ± 57 ms in capsaicin (paired *t*-test, *p* ˂ 0.05). Current–voltage (I-V) relationships of Ca^2+^ currents recorded in the absence and presence of 30 µM capsaicin (*n* = 5) are presented in Figure 5B. The effects of capsaicin (30 µM) were also compared in two different holding potentials (−80 mV versus −50 mV). There was a slight but statistically significant difference between −80 mV and −50 mV holding potentials (Paired *t*-test, *p* < 0.05), suggesting that the effect of capsaicin is weakly voltage-dependent (Figure 5C). A summary of the effects of SB-366791 (10 µM), capsazepine (10 µM), capsaicin (30 µM) + SB-366791, and vanillin (30 µM) on the peak amplitudes of L-type Ca^2+^ currents is presented in Figure 5D. There is no statistically significant difference between capsaicin alone and capsaicin + SB-366791 groups (ANOVA with Bonferroni post hoc test, *p* > 0.05). However, the inhibition by capsazepine is significant (Paired *t*-test, *p* < 0.05).

### 3.3. Docking Studies and Molecular Dynamics Simulations

The docking results for the seven vanilloid compounds are summarized in Table 1. Capsaicinoids, compounds with an aliphatic tail (capsaicin, dihydrocapsaicin, nonivamide, and nordihydrocapsaicin), exhibited the most favorable binding free energies. In contrast, vanilloids, compounds lacking the aliphatic tail (vanillin, vanillyl alcohol, vanillylamine) displayed weaker binding scores and less extensive interactions within the pocket. Notably, the presence of the aliphatic tail correlates with functional inhibition in electrophysiology experiments presented in this study, indicating that the tail is essential for activity at the CaV1.1 allosteric site. This structure–activity relationship highlights the importance of the presence of the aliphatic tail in favorable conformation for both optimal receptor engagement and functional modulation.

All seven compounds (capsaicin, dihydrocapsaicin, nonivamide, nordihydrocapsaicin, vanillin, vanillyl alcohol, and vanillylamine) were subjected to MD simulations in a complex with CaV1.1, starting from the most favorable docked conformation. Trajectories for all compounds showed comparable stability within the binding pocket. RMSD analysis indicated that the receptor backbone remained stable during the 100 ns production runs (RMSD plateau ~2.1 Å), while capsaicinoids and vanilloid compounds exhibited stable accommodation within the pocket (RMSD ~1.5 Å). Clustering analysis identified representative conformations, with the most populated cluster encompassing approximately 80% of simulation frames. These conformations were used for detailed characterization of ligand–receptor interactions.

**Figure 6:**
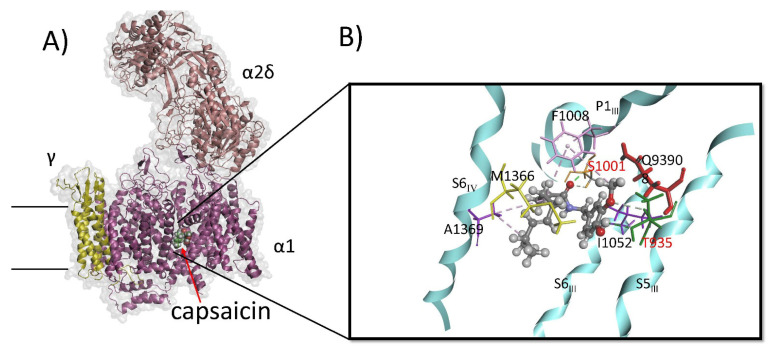
Binding pose and key interactions of capsaicin in CaV1.1. (**A**) CaV1.1 channel (Protein Data Bank ID code 7JPX.pdb) Binding pose of capsaicin within the binding site of the CaV1.1 after molecular dynamics simulations. Capsaicin adopts a bent orientation that fits tightly within the hydrophobic S6–P1 cavity between domains III and IV. (**B**) Specific interactions of capsaicin within the binding pocket. Key residues involved in the interactions include Ser1011 (orange), Phe1008 (pink) on P1_III_ helix; Gln939 (red), Thr935 (green) on S5_III_ helix; Ile1052 (purple) on S6_IV_ helix; and Met1366 (yellow), Ala1369 (purple) on S6_III_ helix.

Molecular dynamics simulations show that all capsaicinoids adopt a similar bent conformation within the CaV1.1 allosteric binding pocket. A representative MD-refined pose of capsaicin, located between helices S5_III_, P1_III_, S6_III_, and S6_IV_, is shown in Figure 6A. The detailed interactions are illustrated in Figure 6B. In this pose, the amide carbonyl oxygen forms a stable hydrogen bond with the side-chain hydroxyl of Ser1011 (orange), part of the P1 helix, helping to anchor the ligand’s central region. Phe1008 (pink), also on the P1_III_ helix, engages capsaicin at two contact points: it packs against the phenolic ring and forms hydrophobic π–alkyl interactions with the aliphatic tail.

The methoxy group of capsaicin forms a polar interaction with Gln939 (red), while the phenolic hydroxyl donates a hydrogen bond to Thr935 on the S5_III_ helix (green). The aliphatic tail bends toward the interior of the cavity, packing optimally against a hydrophobic surface formed by Met1366 and Ala1369 on S6_IV_, which make alkyl–alkyl contacts that stabilize the tail region. This bent arrangement enhances van der Waals complementarity and positions the amide linker toward the more polar portion of the site. Overall, the MD-derived pose indicates that the bent conformation—supported by hydrophobic burial of the tail, π–alkyl stacking with Phe1008, stable hydrogen-bonding interactions, and favorable aromatic packing—facilitates tight and persistent binding of capsaicin at the CaV1.1 binding site.

**Figure 7:**
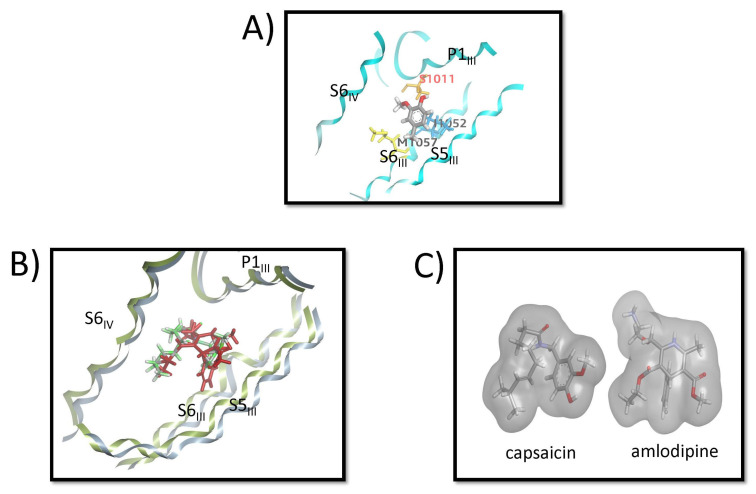
Binding poses and comparison of vanilloids and amlodipine in CaV1.1. (**A**) MD-refined binding pose of vanillyl alcohol within the CaV1.1 binding site. The ligand adopts an orientation between the P1_III_ and S6_III_ helices. Key interactions include a hydrogen bond between the phenolic OH and Ser1011 (orange), hydrophobic contacts between the terminal CH_3_ group and Met1057 (yellow), and a π–alkyl interaction between the aromatic ring of vanillyl alcohol and Ile1052 (blue) on the S6_III_ helix. (**B**) Alignment of the MD-derived pose of capsaicin with the amlodipine structure from 7JPX.pdb, illustrating the overlap in their binding orientations. (**C**) Surface representation comparing the MD-derived capsaicin pose with amlodipine from 7JPX.pdb, highlighting the similarity in overall shape and occupancy of the binding pocket.

All three vanilloids (vanillin, vanillyl alcohol, and vanillylamine) adopt similar binding poses and interaction patterns within the CaV1.1 binding pocket. Figure 7A shows the MD-refined binding pose of vanillyl alcohol, which positions itself between the P1_III_ and S6_III_ helices. Key interactions include a hydrogen bond between the phenolic OH and Ser1011 (orange), hydrophobic contacts between the terminal CH_3_ group and Met1057 (yellow), and a π–alkyl interaction between the aromatic ring and Ile1052 (blue) on the S6_III_ helix. Figure 7B shows the alignment of the MD-derived capsaicin pose with the amlodipine structure from 7JPX.pdb, while Figure 7C presents the corresponding surface comparison. These alignments reveal that the bent conformation of capsaicin closely resembles the shape and spatial occupancy of amlodipine, which may help explain the experimental observation that capsaicin acts as an inhibitor of CaV1.1.

## 4. Discussion

The main finding of this study is that capsaicin and its analogs, but not vanillin and vanillin derivatives, inhibit the function of L-type VDCCs in mammalian skeletal muscle in a TRPV1-independent manner. Earlier investigations have shown that ^45^Ca^2+^ effluxes evoked by high K^+^ are inhibited by dihydropyridine-class Ca^2+^ channel antagonists in a stereo-specific manner, suggesting that ^45^Ca^2+^ effluxes are mediated by L-type Ca^2+^ channels in T-tubule membranes employed in this study [7,26]. Capsaicin-induced release of Ca^2+^ from intracellular stores and subsequent increases in intracellular Ca^2+^ levels have been shown in a variety of cell types (for a review, [20]). Notably, changes in intracellular Ca^2+^ concentrations have been reported to alter functional characteristics of L-type VDCCs [43,44,45]. However, T-tubule membranes do not contain intracellular organelles [26,27]. Thus, it is not likely that the inhibitory effect of capsaicin observed in T-tubule membranes involves alterations of Ca^2+^ levels or the activation of TRPV1 receptors reported to be expressed in the intracellular organelles of the skeletal muscle [41,42].

Capsaicin, in the concentration range used in this investigation (1–100 µM), has been shown to inhibit the function of ligand-gated [21,22] and voltage-gated ion channels [23,24] in a TRPV1-indepenent manner (for a review [20]). In the present study, TRPV1 receptor antagonists SB-366791 did not antagonize the inhibition of L-type Ca^2+^ channels by capsaicin, suggesting that the effects of capsaicin observed in this study do not involve TRPV1 receptors. Interestingly, capsazepine (10 µM), a widely used TRPV1 antagonist, alone directly inhibited L-type Ca^2+^ channels. Similarly, direct inhibitory effects of capsaicin [23,24] and capsazepine [40,46], but not SB-366791 on L-type VDCCs, have been reported earlier [40].

Structure–activity relationship studies on TRPV1 receptors indicate that while vanillyl and amide bond moieties (Figure 1 boxes A and B) are mainly responsible for maintained sensory neuron excitation, the aliphatic tail (Figure 1 box C) provides maximal potency [16,47]. In order to study the structural requirements of capsaicin actions, we have comparatively tested the effects of capsaicin analogs and different vanilloids. Although nonivamide, dihydrocapsaicin, and nordihydrocapsaicin inhibited L-type VDCCs, vanillin, vanillyl alcohol, and vanillylamine did not significantly affect the function of these channels, indicating that unlike TRPV1 receptors, the inhibitory effect of capsaicin on VDCCs does not require a vanilloid moiety and instead hydrophobic tail (Figure 1 box C) significantly contributes to the inhibitory effect of the capsaicin.

As a highly lipophilic compound with a LogP (octanol–water partition coefficient) value of 3.8, capsaicin is likely to first be dissolved in the lipid membrane [48] and subsequently modify the functional characteristics of ion channels either by binding to hydrophobic amino acids of the channels and/or changing the physicochemical features of the membrane. Notably, capsaicin, at the concentration range used in this study, was shown to affect the function of voltage-dependent K^+^ and Na^+^ channels by altering the elasticity of the bilayer [49,50,51] and other physicochemical characteristics of cell membranes such as membrane fluidity [52,53] and dipole potential [54]. However, the direct effects of capsaicin might not be solely attributed to modifications in the biophysical properties of the lipid membrane. Through lateral diffusion in the lipid membrane, capsaicin can reach the hydrophobic binding site(s) on the transmembrane domains of ion channels and modulate their functional characteristics. Capsaicin may therefore directly affect the function of ion channels both by binding to hydrophobic amino acids and by modifying the properties of the lipid bilayer.

The MD-derived poses of capsaicin, dihydrocapsaicin, and nonivamide show notable similarity to the binding mode of amlodipine reported for CaV1.1 (skeletal L-type VDCC) in the cryo-EM structure [32]. In both cases, the ligands occupy the cleft adjacent to the III–IV fenestration, with their aromatic head groups oriented toward the S5–S6 interface and their extended substituents projecting into the hydrophobic cavity. For amlodipine, this role is fulfilled by its aminoethoxymethyl side chain (an elongated, flexible moiety extending from the dihydropyridine core), while for capsaicinoids, the aliphatic chain serves as the primary extended substituent. Capsaicin adopts a bent conformation (Figure 6A,B and Figure 7B,C) that aligns well with the curved geometry of the dihydropyridine ring and attached side chain of amlodipine, allowing both molecules to follow the contour of the fenestration lumen.

Residue-level interactions also reveal parallels between capsaicin and amlodipine. Similarly to dihydropyridine (DHP) antagonists, capsaicin forms hydrogen bonds with Ser1011 (P1) and Thr935 (S5), residues known to participate in ligand anchoring within the fenestration. These polar interactions stabilize the ligand at the S5–S6 interface and are complemented by hydrophobic and π–alkyl interactions with neighboring S6 and *p*-helix residues. The π–alkyl interaction between capsaicin’s aliphatic chain and Phe1008 (P1 helix) resembles the hydrophobic and aromatic contacts formed by the dihydropyridine core of amlodipine. Collectively, the similarity in binding location, ligand shape, and interaction pattern provides a structural rationale for the inhibitory effects of capsaicin, dihydrocapsaicin, and nonivamide on CaV1.1. These compounds appear to occupy a DHP-like binding pose within the fenestration, suggesting that inhibition of L-type channels can arise from a shared set of structural and interaction principles across chemically distinct ligands.

Additional support comes from the observation that capsaicin contacts many of the same residues as amlodipine, including Ser1011, Thr935, Met1366, Phe1008, Ile1052, and Ala1369. Gao and Yan [32] showed that DHP antagonists stabilize a non-conducting, inactivated conformation of CaV1.1 by forming anchoring interactions across the S5–S6 interface. These interactions constrain S6 movement and limit the conformational transitions required for pore opening. Capsaicin’s analogous hydrogen bonds and hydrophobic contacts may contribute to a similar stabilizing effect: the interaction with Ser1011, located toward the intracellular end of S6, may restrict S6 bending and rotation, while the interaction with Thr935 may help maintain an S5 position that disfavors pore dilation.

Although capsaicin reproduces many of the key interaction features of DHP antagonists, its affinity is substantially lower than that of amlodipine, which binds in the nanomolar range. This difference suggests that capsaicin engages the CaV1.1 pocket with reduced specificity and may also interact with additional lower-affinity sites elsewhere in the receptor. Vanilloids lacking the aliphatic tail exhibit even weaker interaction patterns, primarily contacting residues on P1_III_ and S6_III_. This limited set of interactions is consistent with experimental data showing that vanilloid compounds do not inhibit CaV1.1. Overall, the results of docking studies and molecular dynamic simulations indicate that capsaicinoids with an aliphatic tail show stronger binding and stable bent conformations in CaV1.1, forming hydrogen bonds with Ser1011 and Thr935 and hydrophobic/π–alkyl contacts with Phe1008, Ile1052, Met1366, and Ala1369, resembling the binding mode of amlodipine. These interactions support tight receptor engagement and functional inhibition. In contrast, vanilloids lacking the tail displayed weaker binding and limited interactions, consistent with their lack of activity.

The extrapolation of our findings to clinical applications is not straightforward but may have some implications in mitigating the pathological conditions in which L-type VDCCs play a role. To date, splicing errors or mutations in the CACNA1S, the gene encoding for L-type VDCCs, have been associated with four different kinds of muscle diseases [1]. These include VDCC (CaV1.1)-related myopathies, malignant hyperthermia susceptibility, hypo- and normokalemic periodic paralysis, and myotonic dystrophy type 1 [55]. Muscle weakness in patients with myotonic dystrophy has been shown to be associated with increased excitation-coupled calcium entry and the abnormal expression of the Ca^2+^-permeating developmental CaV1.1e splice variant in adult muscle [56,57], potentially leading to chronic Ca^2+^ overload, dysregulation of intracellular Ca^2+^ levels, and mitochondrial damage [11]. Blockers of L-type VDCCs have been suggested to mitigate both myotonic [58,59] and nonmyotonic dystrophies [60,61,62,63,64]. Thus, it is plausible to speculate that the inhibition of L-type VDCCs by capsaicin-like compounds could be a starting point for investigating novel therapeutic strategies for these conditions, particularly those associated with the gain of function mutations of CaV1.1.

In recent years, capsaicin has been used as a dietary supplement to reduce appetite and weight gain. Pharmacokinetic studies suggest that approximately 50–90% of the ingested capsaicin is absorbed through the gastrointestinal tract. However, due to rapid and high-level hepatic first-pass metabolism, only a fraction of capsaicin reaches systemic circulation, resulting in low bioavailability and low peak plasma concentrations at sub-µM levels, with a half-time of 30–60 min [65]. Therefore, a daily intake of capsaicin supplements (1 to 30 mg/day) would not raise blood concentrations to a level that would lead to the significant inhibition of L-type VDCCs observed in this study. However, topical capsaicin application is often employed for analgesia and has been demonstrated to be well-absorbed from skin with a high level of bioavailability [65,66]. The concentration of capsaicin in topical preparations ranges from 3 to 260 mM (0.1–8%). Considering that 2% of topical capsaicin is absorbed through the skin [67], dermal concentrations of capsaicin are likely to be between 0.06 and 5.2 mM for topical preparations containing 0.1% and 8% capsaicin, respectively. Notably, due to its highly lipophilic structure, capsaicin concentrations in cell membranes are likely to be significantly higher than in the extracellular compartment. In animal studies, capsaicin concentration in the spinal cord and brain were approximately 5-fold higher than in plasma following intravenous or subcutaneous administration [66]. Thus, the effect of capsaicin on L-type VDCCs observed in this study may be pharmacologically relevant.

## 5. Conclusions

In this study, using electrophysiological and biochemical methods, it was demonstrated that, in the mid µM concentration range, capsaicin and capsaicin analogs, but not vanillin and vanillin derivatives, directly inhibited depolarization-induced Ca^2+^ effluxes in rabbit transverse tubule membranes and L-type Ca^2+^ currents in rat skeletal muscle myotubes in a TRPV1-independent manner. In addition, in docking studies and molecular dynamic simulations, capsaicinoids with an aliphatic tail showed stronger binding and stable bent conformations in CaV1.1, forming hydrogen bonds with Ser1011 and Thr935 and hydrophobic/π–alkyl contacts with Phe1008, Ile1052, Met1366, and Ala1369, resembling the binding mode of amlodipine. In conclusion, these findings suggest that capsaicin directly inhibits the function of L-type VDCCs in mammalian skeletal muscles in a TRPV1-independent manner.

## Figures and Tables

**Table 1:** Predicted free binding energies of capsaicinoids (compounds 1–4) and vanilloid derivatives (compounds 5–7) with the α1 subunit of the CaV1.1 channel, obtained from docking.

Compounds	Free Energy of Binding, kcal/mol
1. Capsaicin	−6.9
2. Dihydrocapsaicin	−6.8
3. Nonivamide	−6.4
4. Nordihydrocapsaicin	−6.7
5. Vanillin	−4.9
6. Vanillyl alcohol	−5.0
7. Vanillylamine	−4.9

## Data Availability

The data that support the findings of this study are available on request from the corresponding author.

## Supplementary Materials

The following supporting information can be downloaded at: https://www.mdpi.com/article/10.3390/biom16010135/s1, Figure S1: Effects of capsazepine, a TRPVI antagonist, and PN-200-110 (Isradipine), L-type Ca^2+^ channel blocker on L-type Ca^2+^ currents in rat myotubes. Traces of currents recorded before (control) and 5 min after administration of the test compounds were shown.
