# Supplementary material for: Direct Effects of Capsaicin on Voltage-Dependent Calcium Channels of Mammalian Skeletal Muscle
Source: Biomolecules. 2026 Jan 13;16(1):135. doi: 10.3390/biom16010135 (PMC12839150; doi:10.3390/biom16010135)
Supplement: Supplementary file 1 [file biomolecules-16-00135-s001.zip › biomolecules-4063341-supplementary.pdf]

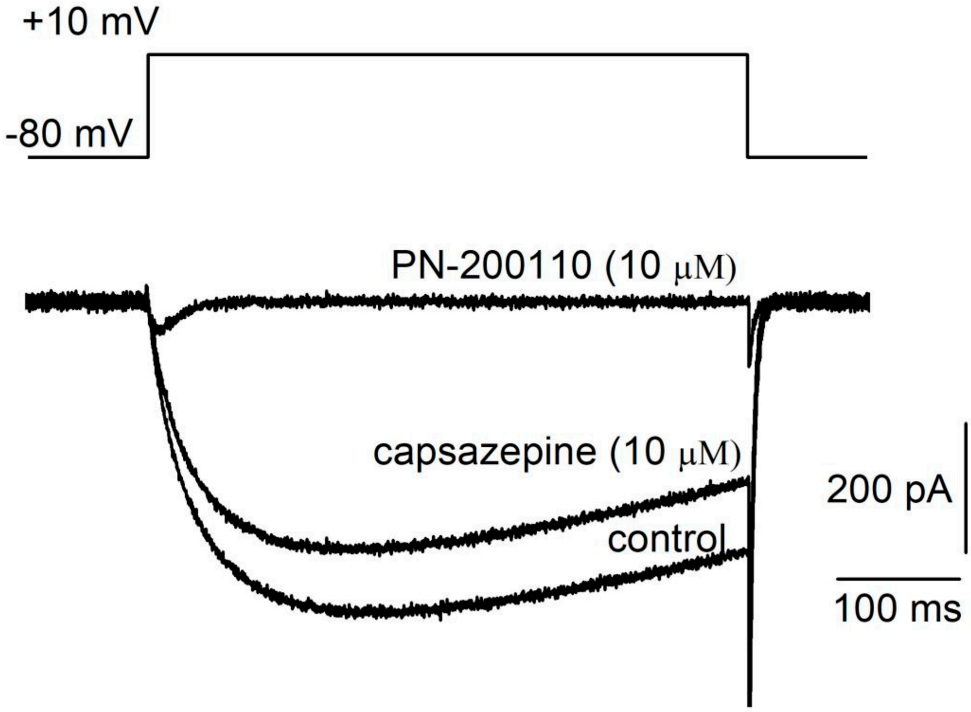

Supplemental Figure S1: Effects of capsazepine, a TRPVI antagonist, and PN-200-110 (Isradipine), L-type Ca^{2+} channel blocker on L-type Ca^{2+} currents in rat myotubes. Traces of currents recorded before (control) and 5 min after administration of the test compounds were shown.
